# Reference based transcriptome assembly of *Piper nigrum L.* reveals novel genes and transcripts in drought tolerance

**DOI:** 10.3389/fpls.2025.1708920

**Published:** 2026-01-21

**Authors:** Sona Charles, Muhammed Fayad Abdulkabeer, K. S. Krishnamurthy, Theertha Azhakoth Parambathu, T. E. Sheeja

**Affiliations:** 1 Division of Crop Improvement and Biotechnology, Indian Council for Agricultural Research (ICAR)- Indian Institute of Spices Research, Kozhikode, India; 2 Division of Crop Production and Post-Harvest Technology, Indian Council for Agricultural Research (ICAR)- Indian Institute of Spices Research, Kozhikode, India

**Keywords:** black pepper, drought, spices, stress response, transcriptome

## Abstract

**Introduction:**

Black pepper (*Piper nigrum* L.), renowned as the “King of Spices,” holds significant economic and medicinal value but is highly susceptible to drought stress, which impacts its growth and productivity. Several studies have reported the impact of drought stress on morphological, physiological and biochemical characteristics, while the molecular mechanism underlying drought tolerance remains largely unexplored.

**Methods:**

This study focusses on the molecular basis of drought tolerance in black pepper through identification of differentially expressed genes (DEGs) by comparative transcriptome analysis involving drought-tolerant Accession (No. 4226) under control and water deficit conditions, and validation of these DEGs by co-expression analysis involving drought-tolerant (IISR Thevam and Acc. No. 4226) and drought-susceptible (Panniyur-1) genotypes under water-deficit conditions.

**Results:**

Reference based assembly of RNAseq data and differential gene expression analysis revealed 2,780 DEGs such as *RUBISCO-S*, *50S_RP*, *SPX*, associated with photosynthetic carbon assimilation, stress-induced regulation of protein synthesis and phosphate homeostasis under nutrient and drought stress, respectively. Functional annotation highlighted enriched biological processes such as metabolic reprogramming and secondary metabolite biosynthesis, while pathway analyses emphasized the role of starch and sucrose metabolism and RNA processing pathways in drought adaptation.

**Discussion:**

Validation of key DEGs such as catalase, defensin, *RUBISCO*, *MYB101*, *SGNH*, *GIB67* and *ZAT10* through RT-qPCR confirmed the transcriptome data and the higher expression in drought tolerant accessions, indicated their involvement in imparting tolerance to drought. The findings also provided valuable insights regarding correlation of molecular and physiological mechanisms underlying drought tolerance in black pepper thereby laying the groundwork for developing high-yielding, drought-tolerant black pepper cultivars.

## 1 Introduction

Black pepper, also known as ‘King of Spices’ is one of the most widely cultivated and traded spices worldwide. In addition to its culinary utility, black pepper also possesses medicinal activities such as anti-cancer, anti-inflammatory, analgesic, anticonvulsant, anti-ulcer, antioxidant, cytoprotective and anti-depressant effects (Butt et al., 2013). It is known for its pungent taste and aroma which is due to the presence of the active ingredient 1-piperoylpiperidine or piperine, whose content is influenced by the place of origin and climatic or drying conditions (Sozzi et al., 2012). Drought and water deficit stress have been identified as major factors affecting black pepper productivity, leading to considerable economic losses (George et al., 2017; Negi et al., 2021) due to its impact on growth, physiology and disease resistance (Krishnamurthy et al., 2016). Black pepper plants can endure temperature ranging from 10°C to 40°C, with the ideal temperature averaging around 28°C. For optimal root growth, the soil temperature should be between 26°C and 28°C and an ideal uniform rainfall of about 2000 mm is required for optimum productivity (Krishnamurthy et al., 2011). Though it prefers a tropical climate with high relative humidity and minimal variation in day length throughout the year, the crop cannot withstand excessive heat and dry conditions (Sivaraman et al., 1999) and is highly susceptible to water scarcity due to their extensive leaf surface and high transpiration rates (Gonzalez-Dugo et al., 2010). The escalating climate crisis has prolonged and intensified drought conditions, has resulted in significant economic losses in black pepper up to 70% (De Pascale et al., 2003).

Black pepper adapts to drought primarily by increasing water use efficiency, intercellular CO_2,_ root penetration and by reducing stomatal conductance, chlorophyll content and stability, leaf area and photosynthesis (Vasantha et al., 1990; Krishnamurthy and Saji, 2006; Prakash et al., 2023). Tolerant varieties showed higher water retention capacity, lower cell membrane permeability and increased thickness of abaxial leaf epidermis along with stomatal density, greater presence of fine roots, thicker periderm and starch accumulation in roots (Krishnamurthy et al., 2000; George et al., 2017; Ferreira et al., 2024). It is also reported that impact of temperature above 40°C coupled with low relative air humidity are detrimental to black pepper (Ambrozim et al., 2022). Though it shows poor responses to rehydration and resilience to long term spells of drought (Ferreira et al., 2024), prior exposure to stress improves the adaptive responses (Ambrozim et al., 2022).

Black pepper exhibits a range of adaptive mechanisms to withstand drought conditions, allowing it to adapt to specific habitats and support their growth and development. These adaptations involve intricate physiological and biochemical responses. During drought stress in black pepper, plant height and leaf area tend to decrease, with leaf expansion slowing down even before the soil reaches critical moisture deficiency (Ramadasan and Vasantha, 1994). Some black pepper accessions have been observed to have an increased root to shoot ratio compared to others under water-limited conditions (Krishnamurthy et al., 2016). In black pepper severe water stress leads to a reduction in chlorophyll a, chlorophyll b, and total chlorophyll content (Krishnamurthy et al., 2016), while leaf wax deposition increases as a protective measure (Thankamani et al., 2002), and the total amino acid levels rise in response to drought (Krishnamurthy and Ankegowda, 1998). In water-deficient black pepper plants, physiological functions such as photosynthetic rate (A), stomatal conductance (gs), gas exchange, and transpiration are significantly impacted. Water stress also alters enzymatic activity, reducing catalase and acid phosphatase levels while enhancing peroxidase and polyphenol oxidase activity (Krishnamurthy et al., 2000; Thankamani et al., 2003) also reported decreased acid phosphatase activity declines and lipid increased peroxidation in water stress black pepper plants.

Transcriptomics have paved way to identification of important genes and pathways related to drought providing a theoretical guidance for strategies for early selection of drought responsive genotypes as well as for developing future transgenics for drought tolerance in many crops (Mir et al., 2012). Several transcriptome studies have been conducted in black pepper, providing valuable insights into key genes and pathways associated with various traits, including quality, disease resistance, and stress tolerance (Hu et al., 2015; Babu Paul et al., 2019; Lau et al., 2020; Sreekumar et al., 2022; Das et al., 2025). In another study by George et al., 2017 (George et al., 2017), though a set of genes involved in drought tolerance could be identified and validated by qRT-PCR, these genes were randomly chosen from the *P. nigrum* transcriptome challenged with *Phyphthora* based on a *de novo* approach. At present high fidelity sequences and whole genome sequence data is available in black pepper (Hu et al., 2019), based on which a reference genome-based approach, that enhances the accuracy of transcript quantification and variant analysis was adopted by us for a comparative transcriptome analysis under drought stress condition *per sey* along with validation of key candidate genes through qPCR-based expression profiling, as a direct experimental support to our computational findings. This comprehensive approach not only builds upon previous findings but also provides a deeper understanding of the genetic and molecular basis of drought response in black pepper.

Differential Transcriptome analysis in the drought tolerant accession 4226 led to identification of important genes, transcription factors and pathways associated with stress tolerance (Negi et al., 2021). The study also identified SSRs, SNPs and InDels and catalogued the available genomic information into a database for future use in improving drought resilience in black pepper. However, the *de novo* approach adopted as well as lack of validation of the identified key DEGs limits further exploitation of these genomic resources. While (Negi et al., 2021) utilized the same transcriptomic dataset to perform a *de novo* assembly-based gene mining approach, their primary focus was on identifying genes associated with drought stress and creating a web-based genomic resource. In contrast, our study adopts a more integrative and mechanistic perspective, leveraging the available reference genome to perform reference-guided alignment, enabling higher precision in transcript quantification. We go beyond simple gene discovery by conducting a comprehensive differential expression analysis, identifying key DEGs, annotating biological processes and metabolic pathways, and highlighting gene networks potentially involved in drought tolerance. Additionally, our study uniquely includes comparative transcriptome analysis between a drought-tolerant (Accession 4226) and susceptible (Panniyur 1) genotype, which provides a functional understanding of genotype-specific responses. Furthermore, we validated the expression of selected DEGs using qPCR, strengthening the biological relevance of our findings. Therefore, while the earlier work laid a foundational resource, our research offers deeper insights into the molecular mechanisms underlying drought tolerance in black pepper.

Our study presents a comprehensive transcriptome analysis of drought tolerant black pepper variety, Accession 4226 under control and drought stress conditions and validation of important DEGs in tolerant and susceptible genotype under induced drought stress. We aimed to elucidate the molecular mechanism underlying the response of black pepper to drought stress, which may eventually help in devising strategies for molecular breeding for drought resistance. Additionally, we identified the most prevalent genetic variations that serve as crucial markers for studying genetic diversity and trait associations related to drought tolerance in black pepper and also for developing transgenics and gene edited lines tolerant to drought.

## 2 Materials and methods

### 2.1 Plant materials and growing conditions

All plant materials used in this study were sourced from ICAR–Indian Institute of Spices Research, Calicut, India (11°15′N; 75°46′E). For transcriptome analysis, *Piper nigrum* accession 4226 a drought-tolerant genotype identified through field screening (Krishnamurthy et al., 2016) was used. Rooted cuttings were grown individually in pots containing a soil: cow dung: sand mixture (1:1:1) under greenhouse conditions with temperatures ranging from 30–33°C, relative humidity of 70–90%, and a natural 12 h photoperiod. Plants were maintained at field capacity (20–21% soil moisture) through daily irrigation. Drought stress was induced by withholding irrigation for 18 days, reducing soil moisture to 8–9% (Vijayakumari and Purthur, 2014). Leaves from control and drought-treated plants were immediately frozen in liquid nitrogen for RNA extraction and transcriptome sequencing.

For physiological and biochemical analysis, leaves were collected from six-year-old, field grown 4226 plants that experienced one month of natural drought stress (11–12% soil moisture) during the flowering stage, along with fully irrigated control plants maintained at 19–20% soil moisture.

For qPCR validation of differentially expressed genes, four-leaf stage cuttings of two contrasting *P. nigrum* varieties: IISR Thevam (drought-tolerant) and Panniyur 1 (drought-susceptible), were grown in bags under greenhouse conditions (28–33°C, 70–85% RH, natural light). Drought stress was imposed by withholding irrigation, while control plants were regularly watered. Leaf samples were collected at 1, 5, 10, 15, and 18 days after stress treatment, rinsed with RNase-free water, flash-frozen in liquid nitrogen, and stored at −80°C until further analysis.

### 2.2 RNA isolation and library preparation

All the materials used for RNA isolation was soaked and washed with DEPC treated water. Total RNA was extracted using Spectrum Plant Total RNA Kit (Sigma Aldrich, US). The purity and quantity were assessed using a Nanodrop spectrophotometer (DeNovix DS-11). The cDNA synthesis was carried out using Thermo Scientific RevertAid First Strand cDNA Synthesis Kit.

### 2.3 Pre-processing of raw reads and transcriptome assembly

The transcriptome analysis of black pepper accession 4226 induced with drought and a control was performed to identify differentially expressed genes (DEGs). Reference genome sequences already available in black pepper (Hu et al., 2019) as well as the resequencing data available in black pepper accessions (Das et al., 2025) were used for the analysis. Raw RNA-Seq data in FASTQ format were subjected to quality control using FMultiQC (version 0.11.9). FastQC assessed the overall quality of the data by generating metrics such as per-base sequence quality, GC content and adapter contamination. To ensure clean reads for downstream analysis, adapters and low-quality bases were removed using Trim Galore (version 0.6.7) which combines Cutadapt and FastQC functionalities. Trim Galore was run with default parameters and a quality score cutoff of 30. High-quality reads were aligned to the *Piper nigrum* reference genome (accessed on June 2025) using STAR (version 2.7.10a), a splice-aware aligner. STAR was configured with default parameters for genome-guided alignment, allowing the mapping of exon-spanning reads with high accuracy. Mapped reads were quantified to generate gene-level expression counts using HTSeq-count (version 0.13.5). The analysis was performed in the union mode with strandedness set to “no,” producing a matrix of raw counts corresponding to each gene.

### 2.4 Identification of differentially expressed genes

Differential gene expression analysis between accession 4226 and the control was conducted using the R package EdgeR (version 3.40.0). Genes with a false discovery rate (FDR) < 0.05 and a |log2 fold change| ≥ 1 were considered significantly differentially expressed. Normalization of raw counts was performed using the trimmed mean of M-values (TMM) method to account for library size differences.

### 2.5 Functional annotation of transcripts

Functional annotation of the differentially expressed transcripts was performed to assign biological meaning to their functions. The transcript sequences were annotated using BLASTx (version 2.13.0) against the NCBI non-redundant (NR) protein database with an e-value threshold of 1e-5. This provided putative protein functions by identifying homologous sequences. Additionally, STRINGdb was used to obtain Gene Ontology (GO) terms for molecular function, biological process and cellular component categories. To understand the biological pathways associated with the DEGs, the sequences were mapped to the Kyoto Encyclopedia of Genes and Genomes (KEGG) database using the KEGG Automatic Annotation Server (KAAS). Enzyme codes (EC numbers) were assigned to the genes and KEGG pathways were reconstructed to identify significant metabolic and regulatory pathways. Protein family classification and domain prediction were carried out using the InterProScan tool (version 5.59). InterProScan integrated multiple databases, including Pfam, SMART and TIGRFAMs, to identify conserved domains and motifs, offering insights into the structural and functional properties of the transcripts. Functional enrichment analysis of DEGs was performed which identified overrepresented GO terms and pathways, highlighting the key biological processes influenced by the experimental conditions. A p-value cutoff of 0.05 was applied for enrichment significance. In order to understand various sub-categories of GO terms in stress, we performed GO enrichment of exclusively stress related DEGs.

### 2.6 Identification of Cis-elements

Retrieved the 2.0-kb upstream sequences from the start codon (ATG) of each candidate gene in the black pepper genome to identify cis-regulatory elements within their promoter regions. The prediction of these elements was carried out using the PlantCARE database (https://bioinformatics.psb.ugent.be/webtools/plantcare/html/, Data accessed in 12 June 2025) and visualised these using ChromoMap tool (https://cran.r-project.org/web/packages/chromoMap/index.html)

### 2.7 Identification of SNP markers

Single Nucleotide Polymorphisms (SNPs) represent a single nucleotide variation at a specific position in the genome, encompassing both transitions and transversions. In this study, SNPs were detected using freeBayes, a widely employed variant calling tool capable of identifying polymorphisms from aligned sequencing data. FreeBayes was run with specific parameters optimized for sensitivity and specificity, ensuring accurate detection of SNPs. To further minimize the error rate, stringent filtering criteria were applied to the raw SNP calls. Only those SNPs with a sequencing depth (DP) greater than 20 and a mapping quality score higher than 20 were retained. These thresholds ensure high-confidence variant calls by excluding low-quality and ambiguous reads, which are often sources of false positives in SNP detection pipelines. The identified SNPs were subsequently subjected to functional annotation using snpEff, an efficient tool for predicting the potential effects of genetic variations. Annotation includes classifying the SNPs based on their genomic location (exonic, intronic, intergenic, etc.), functional impact (synonymous, non-synonymous, missense, nonsense) and association with coding regions or regulatory elements. To enhance the reliability and interpretability of the results, Variant Effect Predictor (VEP) from Ensembl was also used as a supplementary tool for SNP annotation. VEP provides comprehensive insights into the biological implications of SNPs by integrating data from multiple databases. The combination of high-confidence SNP detection and robust annotation highlights the functional relevance of these variations, particularly in the context of genomic studies and trait mapping. This approach enables the identification of markers linked to key agronomic traits, facilitating genetic improvement programs and offering insights into the molecular basis of trait variation.

### 2.8 Anatomical changes during drought stress

Variations in stomatal aperture size in control as well as 14 DAS (days after stress) were observed using a Leica compound microscope (×400) (scale bars = 20 µm).

### 2.9 Physiological responses under drought stress

Stomatal density (SD) was determined using the rapid leaf impression technique as outlined by Reich (1984) (Reich, 1984), with observations made on three leaves per treatment under a Leica compound microscope fitted with a digital camera. Relative water content (RWC) of the leaves was determined using the protocol described by González and González-Vilar (2001) (González and González-Vilar, 2001). Proline content (PRO) in the leaf tissue was quantified in mg/g following the method of Bates et al. (1973) (Bates et al., 1973). Cuticular wax (CW) content was estimated in mg/g of leaf tissue according to the procedure of Schreiber and Schönherr (1993) (Schreiber and Schönherr, 1993). Chlorophyll a, chlorophyll b, total chlorophyll, and carotenoid (Cx+c) concentrations were analyzed following the protocols of Shabala et al. (1998) and Lichtenthaler (1987) (Lichtenthaler, 1987; Shabala et al., 1998). Total soluble sugar content was measured using the method developed by Karkacier et al. (2003) (Karkacier et al., 2003). Ascorbate peroxidase (APX; EC 1.11.1.11) activity was determined following the procedure of Nakano and Asada (1981) (Nakano and Asada, 1981), while glutathione reductase (GR; EC 1.6.4.2) was assayed according to Carlberg and Mannervik (1985) (Carlberg and Mannervik, 1985). Malondialdehyde (MDA) levels, indicative of lipid peroxidation, were estimated using the thiobarbituric acid (TBA) method as described by Verma and Dubey (2003) (Verma and Dubey, 2003). The concentration of (+)-cis-trans-abscisic acid (ABA) in leaf extracts was measured using the approach reported by Quarrie et al. (1988) (Quarrie et al., 1988). Total glutathione (tGSH) was quantified based on the method of Griffith (1980) (Griffith, 1980). Finally, the activity of monodehydroascorbate reductase (MDHAR; EC 1.6.5.4) was evaluated using the protocol outlined by Hossain et al. (2011) (Hossain et al., 2011).

### 2.10 Validation of DEGs using RT-qPCR reactions

For validation, differentially expressed genes in various stress related processes were selected based on their FPKM values. The upregulated genes included ribulose-1,5-bisphosphate carboxylase small subunit (*RUBISCO-S*), 50S ribosomal protein L27 (*50S-RP*), SPX domain-containing protein 3-like (*SPX*), catalase isozyme 1 (*C-isozyme1*) and defensin J1-2-like (*Defensin-J1)*. Meanwhile, the downregulated genes consisted of Ribulose-1,5-bisphosphate carboxylase (*RUBISCO*), transcription factor MYB101-like (*MYB101*), *SGNH* hydrolase-type esterase domain-containing protein (*SGNH*), hypothetical protein *GIB67*_010252 (*GIB67*) and zinc finger protein *ZAT10*-like (ZFP).

**Figure 1:**
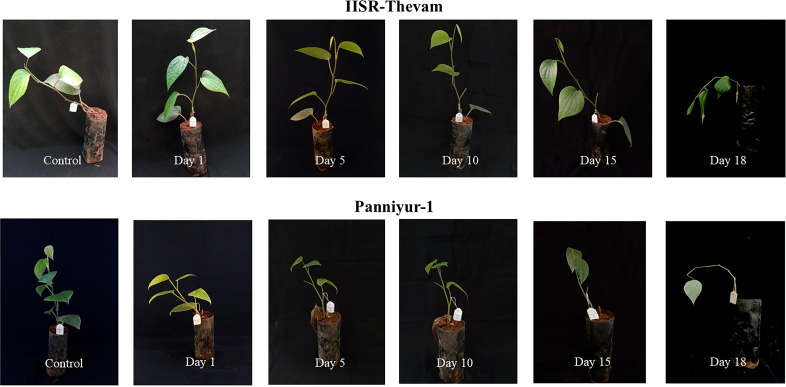
Test and control plants of IISR-Thevam & Panniyur-I.

The validation of expression analysis of key DEGs were conducted in four-leaf stage cuttings of the drought-tolerant variety IISR Thevam and the drought-susceptible variety Panniyur 1 maintained in grow bags within the greenhouse facility at temperatures ranging from 28–33°C under natural light conditions. Leaf samples from both control and water stressed plants were collected on days 1, 5, 10, 15 and 18 of the treatment (**Figure 1**). The collected leaves were rinsed with RNase-free water and immediately frozen in liquid nitrogen and stored at −80°C for further analysis. The experimental setup consisted of three replicates per treatment.

Real-time analysis was conducted using the Rotor-Gene Q apparatus from QIAGEN with the QuantiFast SYBR Green PCR kit (Qiagen). The PCR reaction, in a total volume of 10 μl, comprised 100 ng of cDNA, 5 µl of 2X SYBR Green and 0.5 µM each of forward and reverse primers. The reaction followed a temperature protocol of 5 minutes at 95°C, succeeded by 40 cycles of 10 seconds at 95°C, 30 seconds at 60°C and 20 seconds at 72°C. Post the 40 cycles, specificity of the amplifications was verified by heating the samples from 60°C to 95°C. For each sample 3 technical replicates were employed and the Ct values were used for the analysis. No Template Control (NTC) and No Reverse Transcriptase Control (NRT) were tested for each primer pair to rule out non-specific amplification and the presence of residual genomic DNA in the sample. Actin gene 5’ ACATCCGCTGGAAGGTGC 3’ (F), 5’ TCTGTATGGTAACA TTGTG 3’ (R) was used as the internal control. The primers used for the analysis are listed in **Supplementary Table 1**. The relative gene expression was calculated using the 2^−DDCt^ method (Livak and Schmittgen, 2001).

## 3 Results

### 3.1 Physiological responses of stress

Black pepper accession 4226 exhibited significant physiological and biochemical changes in response to drought stress (**Table 1**). After 18 days of water deficit, relative water content (RWC) declined from 94.2% to 80.0%. Stomatal density remained high (61.2 stomata/10x field).

**Table 1:** Physiological and biochemical parameters of control and water stress induced plants.

Accession 4226	Control	28 days after stress (DAS)
Petiole length (cm)	3.8	
Internodal length (cm)	6.0	
stomata (10x)	61.2	
Epicuticular wax (µg/cm2	3.7	
RWC (%)	91.2a	80.0b
Proline (ug/g)	65.3a	151.5b
Total chl (mg/g)	2.7a	2.1b
Chla/chl b	3.3a	4.2b
MDA (ug/g)	21.3a	53.8b
Total sugars (%)	1.5a	1.7b
Carotenoids (mg/g0	1.0a	0.8b
ABA (ppb)	23.5a	29.8b
GST activity	41.0a	822.0b
MDHAR	87.0a	103.3b
Ascorbate peroxidase	696.6a	675.0b
Glutathione reductase	232.6a	72.6b

### 3.2 Anatomy of leaf stomata

**Figure 2:**
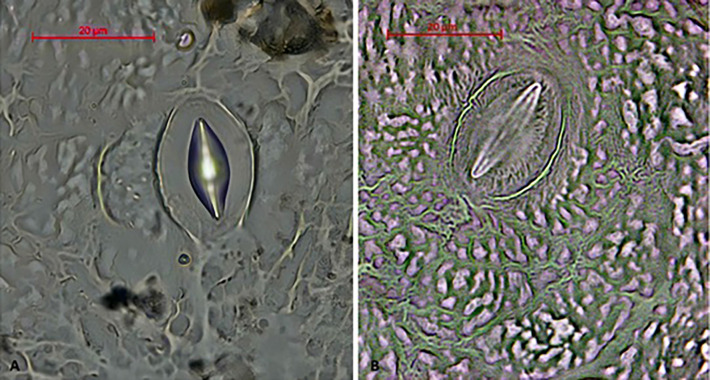
Variations in stomatal aperture size at corresponding stress levels **(A)** Control **(B)** 14 DAS. The image taken observed using a Leica compound microscope (×400) (scale bars = 20 µm).

Accession 4226 exhibits a stomatal frequency of over 70 per 204 mm². While stomatal length remains unchanged following drought treatment, stomatal width decreased compared to the control (**Figure 2**).

### 3.3 Biochemical responses of stress

At the end of 18 DAS, proline content increased from 65.3 to 151.5 µg/g. Similarly, abscisic acid (ABA) levels rose from 23.5 to 29.8 ppb. A notable increase in malondialdehyde (MDA) levels (21.3 to 53.8 µg/g) was observed. Antioxidant enzyme activity showed differential responses. Glutathione S-transferase (GST) activity increased significantly from 41.0 to 822.0 units. Meanwhile, monodehydroascorbate reductase (MDHAR) activity rose from 87.0 to 103.3 units, while ascorbate peroxidase (APX) activity remained relatively stable (696.6 to 675.0 units). However, glutathione reductase (GR) activity decreased from 232.6 to 72.6 units. Photosynthetic pigments were affected, with total chlorophyll content decreasing from 2.7 to 2.1 mg/g, while the chlorophyll a/b ratio increased from 3.3 to 4.2. Carotenoid levels also declined slightly from 1.0 to 0.8 mg/g. These findings (**Table 1**) suggest that black pepper accession 4226 employs a combination of osmotic adjustment, antioxidant defense mechanisms and hormonal regulation, to cope with drought stress.

### 3.4 Illumina paired end sequencing

Paired-end sequencing of drought-induced and control RNA samples was performed using the Illumina HiSeq 2000 platform, with library preparation carried out using the TruSeq RNA Sample Prep Kit v2, generating 101 bp read lengths. The FASTQ files from all samples used in the transcriptome analysis have been deposited in the Sequence Read Archive (SRA) at the National Center for Biotechnology Information (NCBI) under BioProject PRJNA515366, with BioSamples SAMN10754251 and SAMN10754252.

### 3.5 Bioinformatics analysis

The raw reads in FASTQ format were processed using MultiQC, which is designed to provide quality control checks on raw sequence data from high-throughput sequencing pipelines. The control and drought samples consisted of 32772362 and 29735278 sequences respectively with a GC content of 46%. The detailed statistics of the samples are provided in **Table 2**.

**Table 2:** Quality control and read statistics of transcriptome.

Sample	Total sequences	Percentage of duplications	Sequence length	Percentage of GC content	MSeqs	Q30
Control	32772362	45	99	46	16.5	40
Drought-induced	29735278	43	99	46	20	40

The reads were mapped against the reference genome of Black pepper to assess their alignment and coverage. The mapping process revealed high-quality alignments for both control and drought-induced samples. The mapping results confirmed the quality of the sequencing data and its suitability for further downstream analyses. The mapping statistics are provided in **Table 3**.

**Table 3:** Statistics of aligned paired-end reads to the reference genome.

Sample	Number of Reads	Number of Mapped Reads	Mapping percentage
Control	16,386,181	13,665,261	83.4 %
Drought-induced	19,867,639	16,729,004	84.2 %

### 3.6 SNP and variant analysis

The SNP analysis revealed significant genetic variations between control and drought-induced conditions (**Table 4**). The total number of SNPs increased from 143,787 in control plants to 204,464 under drought conditions. A substantial increase in upstream (29,748 to 43,023) and downstream (27,587 to 29,122) variants was observed, highlighting potential regulatory modifications that may influence gene expression. The number of exonic mutations also increased, with stop-gain variants rising from 428 to 572 and stop-loss variants increasing from 146 to 182. Additionally, synonymous SNPs increased from 28,009 to 42,152, while missense variants showed a notable rise from 29,982 to 46,667. The number of intronic SNPs increased from 16,339 to 26,460, along with an increase in splicing variants from 279 to 387. Furthermore, intergenic SNPs increased from 4,842 to 6,773.

**Table 4:** Variant analysis of control and drought induced samples.

Variant		Control	Drought- induced
Upstream		29748	43023
Exonic	Stop gain	428	572
	Stop loss	146	182
	Synonymous	28009	42152
	Non-synonymous		
Intronic		16339	26460
Splicing		279	387
Downstream		27587	29122
Missense variant		29982	46667
Intergenic		4842	6773
Total		143787	204464

### 3.7 Differential expression analysis

**Figure 3:**
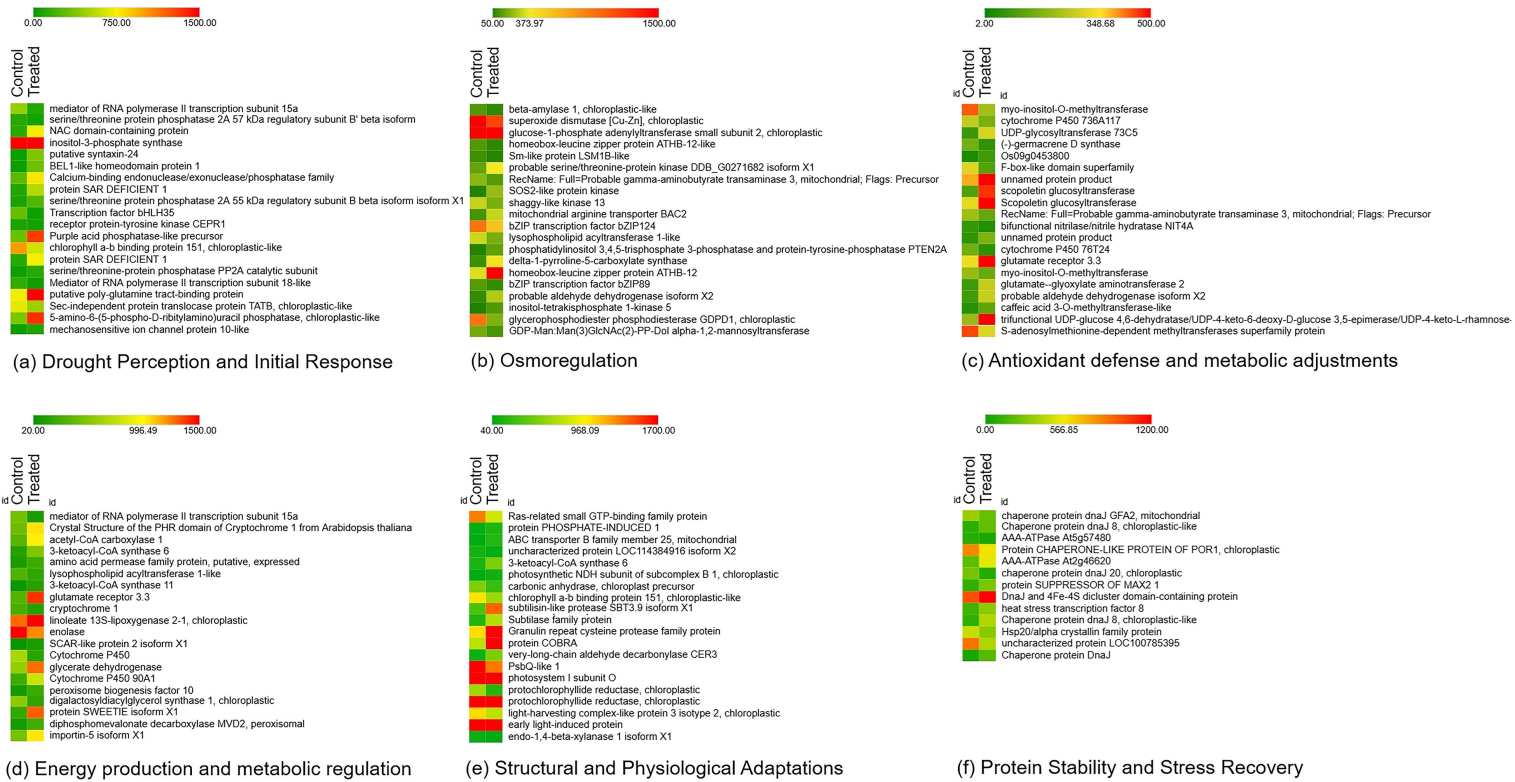
Heatmap of top 20 genes in various processes of drought stress. **(a)** Drought perception and initial response, **(b)** Osmoregulation, **(c)** Antioxidant defense and metabolic adjustments, **(d)** Energy production and metabolic regulation, **(e)** Structural and physiological adaptations **(f)** Protein stability and stress recovery.

Ht-Seq provided a comprehensive measure of gene expression levels, facilitating subsequent differential expression analysis. EdgeR provided statistical routines for determining differential expression in digital gene expression data using a model based on the negative binomial distribution. Genes with an adjusted p-value < 0.05 and |log2 fold change| > 1 were deemed significantly differentially expressed. A total of 3472 genes were found to be expressed in the samples out of which 1512 and 1960 number of genes were upregulated and downregulated respectively (**Figure 3**).

### 3.8 Functional annotation of differentially expressed genes

Taxonomic annotation of the identified differentially expressed sequences revealed a diverse distribution across multiple taxa. Most annotated sequences belonged to streptophyta. Within this group, notable classifications included fabids (558) and liliopsida (444). Additionally, specific orders such as brassicales (73), poales (81), asterids (95) and eukaryota (15). A total of 214 sequences remained unclassified and were grouped as “others,” indicating the presence of novel or less-characterized sequences. This taxonomic distribution suggests that the annotated sequences are predominantly derived from higher plants, reinforcing their relevance to plant genomic studies.

**Figure 4:**
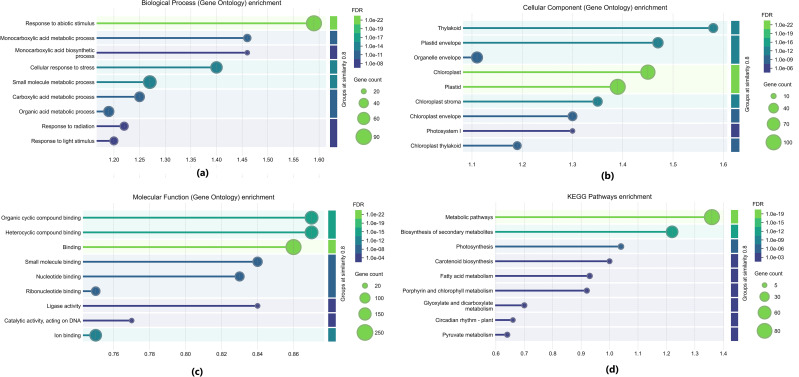
Gene Ontology and KEGG Pathway analysis of DEGs **(a)** Biological Process **(b)** Cellular Component **(c)** Molecular Function **(d)** KEGG Pathways.

The DEGs were classified into the three categories of gene ontology terms, biological process, cellular component and molecular function. Out of all the DEGs, 1478 were not annotated to any GO terms. The functional annotation of transcripts revealed a total of 7,460 Gene Ontology (GO) terms. The plasma membrane (GO:0005886), nucleus (GO:0032991) and cytosol (GO:0005829) were the most enriched cellular component categories, highlighting the functional significance of these compartments in stress response and regulatory mechanisms. Other key components included plasmodesmata (GO:0009910), which are essential for intercellular communication, and chloroplast-related annotations (GO:0009570, GO:0009507, GO:0009941), reinforcing the role of photosynthetic machinery under drought stress. Additionally, mitochondrial functions (GO:0005739) and organelle sub-compartmentalization (GO:0009535) suggest active metabolic and energy-associated processes. In the biological process category the genes were mostly enriched in heat, cold, salinity, water deprivation stress related processes (GO:0009414; GO:0005773; GO:0009651; GO:0009409; GO:0009408) and absicissic acid (GO:0009737). Under the molecular function category of, heterocyclic compound binding (GO:0042802), mRNA binding (GO:0006446), DNA-binding transcription factor activity (GO:1901363) homodimerization activity (GO:0042803) were the processes with highest gene enrichment. Out of the differentially expressed genes, 440 genes were annotated to the category of stress. (**Figure 4**).

### 3.9 Pathway analysis of differentially expressed genes

To comprehensively understand the active biological pathways in differentially expressed genes (DEGs) of Black Pepper Accession 4226 under drought conditions and to identify key stress-related pathways, KEGG pathway enrichment analysis was performed based on the expression profile. The results indicated that 1,402 DEGs were annotated to 1,034 metabolic pathways in the comparison between control and drought-treated samples of Black Pepper Accession 4226. Among these, 616 upregulated DEGs were associated with 543 metabolic pathways, while 786 downregulated DEGs were linked to 645 metabolic pathways.

### 3.10 Transcription factor analysis of differentially expressed genes

**Figure 5:**
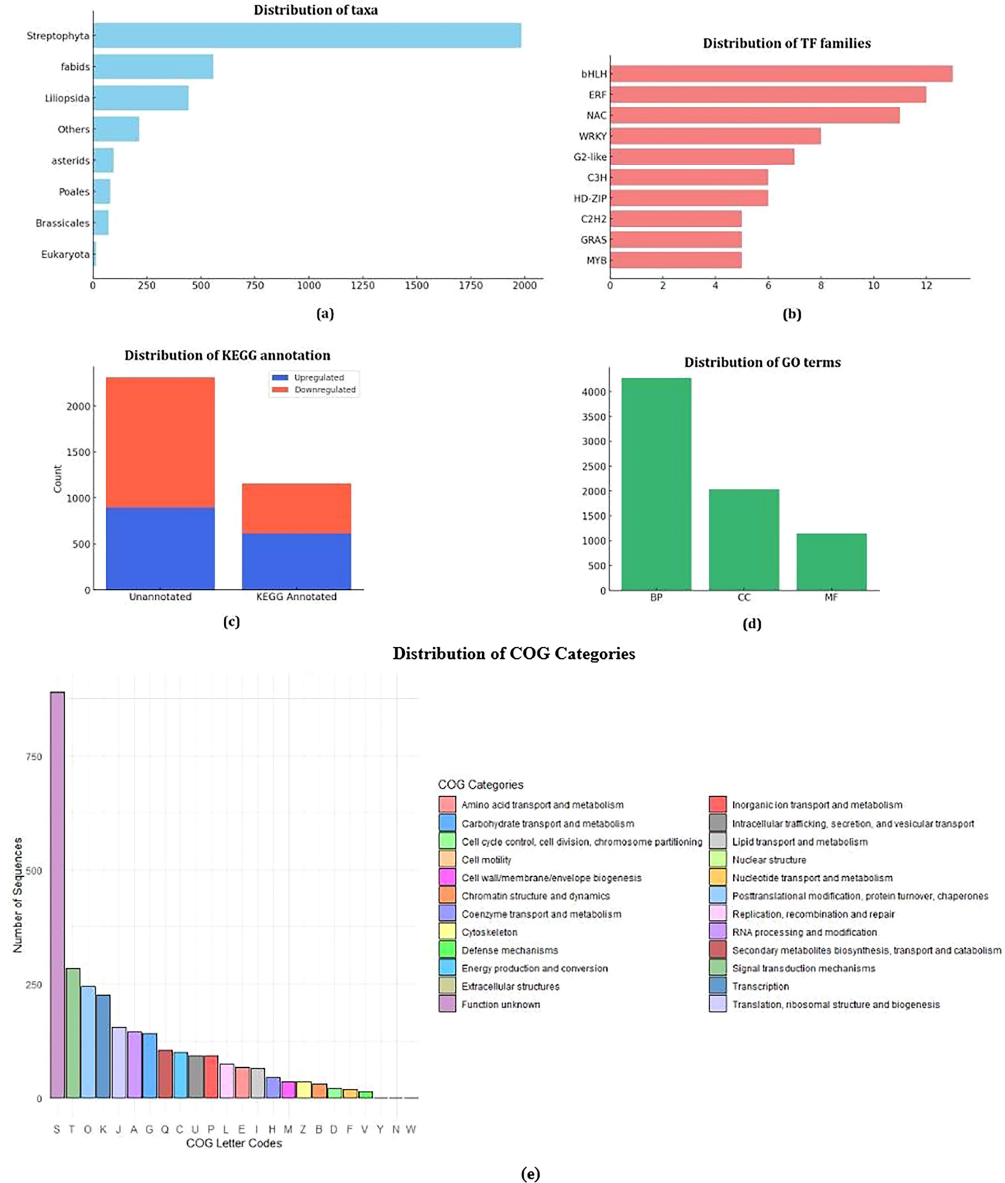
Distribution of various parameters in the transcriptome **(a)** Taxonomy of DEGs **(b)** Transcription Factor Families **(c)** KEGG annotation **(d)** Number of GO Terms **(e)** COG Categories.

Transcription factors (TFs) play a crucial role in plant stress tolerance by acting as upstream regulators that modulate gene expression in various metabolic pathways. We also analyzed the expression profiles of TFs across different processes to assess the complexity of the drought signaling pathway network. A total of 139 genes encoding putative TFs were identified. Five transcription factor (TF) families accounted for 36% of the identified groups, with *bHLH* (13 genes), *ERF* (12 genes), *NAC* (11 genes), *WRKY* (8 genes) and *G2-like* (7 genes) playing key roles in enhancing drought stress tolerance. 52 transcription factors were upregulated while 86 transcription factors were downregulated. Top five downregulated transcription factors belonged to *MYB*, *C2H2*, *ERF*, *RAV* and *MYB* families while top upregulated TFs belonged to *bHLH*, *E2F/DP*, *LBD*, *CO-like*, *ERF* families (**Figure 5**).

### 3.11 Interaction between stress related genes

**Figure 6:**
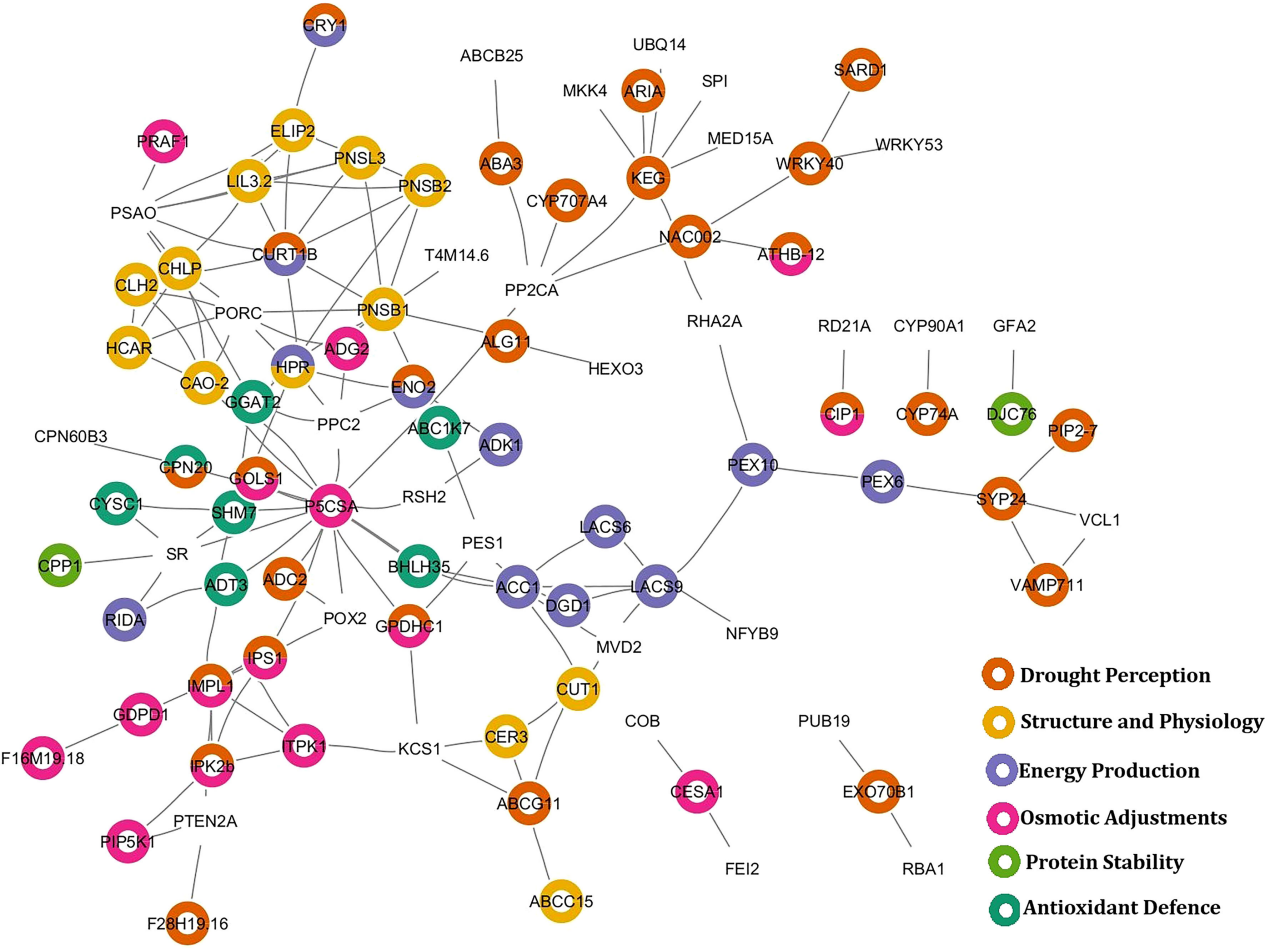
STRING mapping of DEGs in drought stress and their characteristic roles in various physiological processes in drought.

To elucidate the role of genes in regulating the drought response at the protein level, a protein-protein interaction (PPI) network was inferred from the identified DEGs by mapping them to their known or predicted protein products using interaction databases. This approach integrated data from text mining, experimentally validated interactions, and co-expression networks, with the default median confidence level applied to ensure interaction reliability. The homologs of potential DEGs were identified from the Arabidopsis database and analyzed using the STRING database. The network consisted of 138 number of nodes, 140 edges with average node degree 2.03, average local clustering coefficient 0.378 and protein-protein interaction enrichment p-value of 1.11e-16 (**Figure 6**).

### 3.12 Cis-acting elements in promoter regions

**Figure 7:**
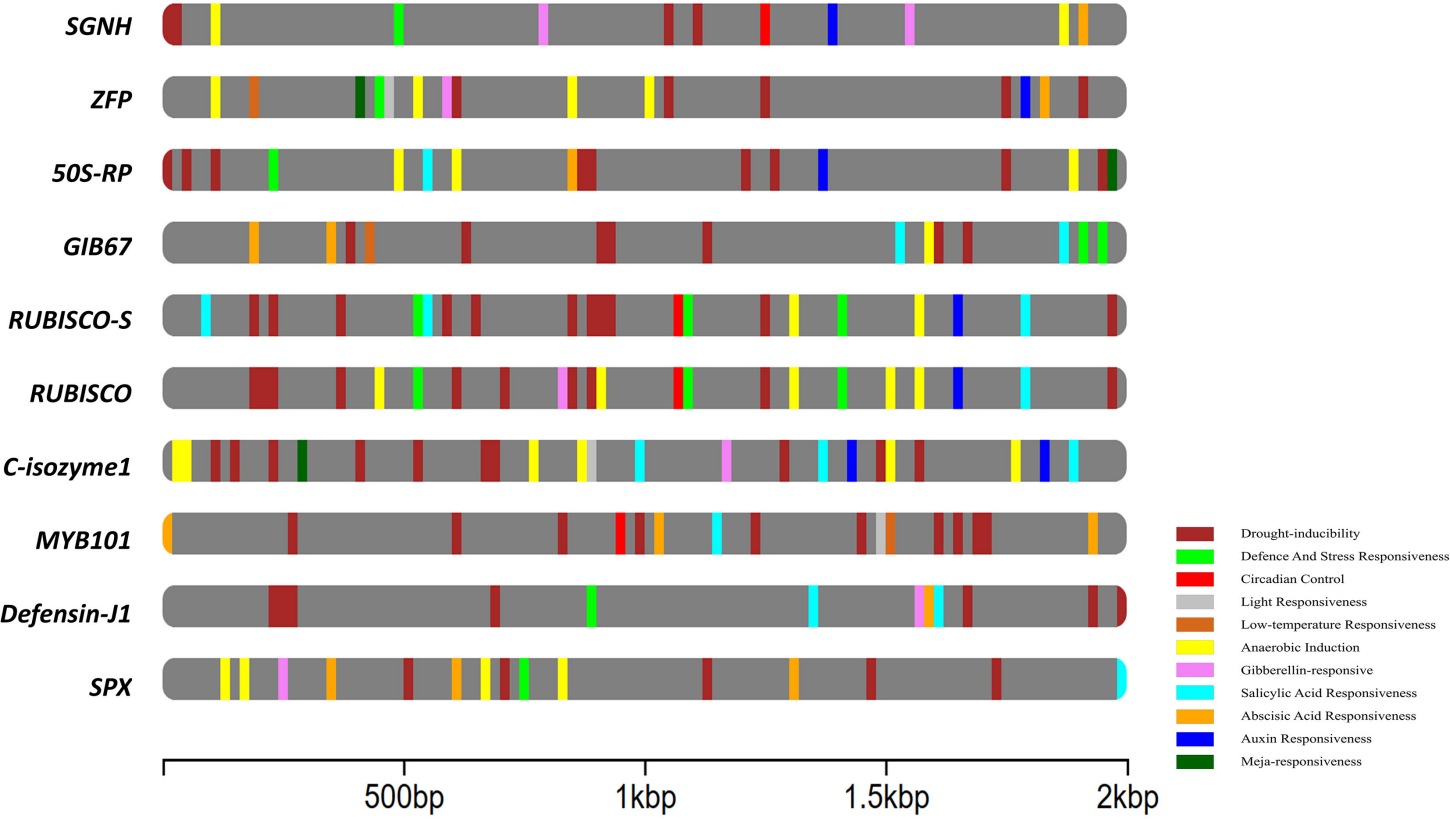
Cis-acting elements identified in the 2kb upstream region of the different genes.

To investigate the regulatory landscape of candidate genes under stress and hormonal conditions, a comprehensive analysis of cis-acting elements in the 2.0-kb upstream promoter regions was performed. As shown in the **Figure 7** and **Supplementary Table S2** various cis-acting elements are predicted in relation with plant stress and hormone responses. Among the stress-related elements, light responsiveness was the most abundant, with 102 elements distributed across all genes, followed by anaerobic induction (27) and low-temperature responsiveness (3). *50s_r_prtn* and *RUBISCO* showed the highest number of light-responsive elements (14 and 13, respectively). *RUBISCO_S*, and *GIB67* showed notable counts of anaerobic induction-related elements (5–6 each), indicating potential roles in hypoxia or water stress adaptation. In hormone-related elements, MeJA-responsiveness was the most enriched with 34 elements, followed by abscisic acid and auxin-responsive elements (13 each). The gene *GIB67* contained the highest number of hormone-responsive elements (8 MeJA-related elements), indicating its possible role in stress hormone signalling.

### 3.13 Validation of DEGs

**Figure 8:**
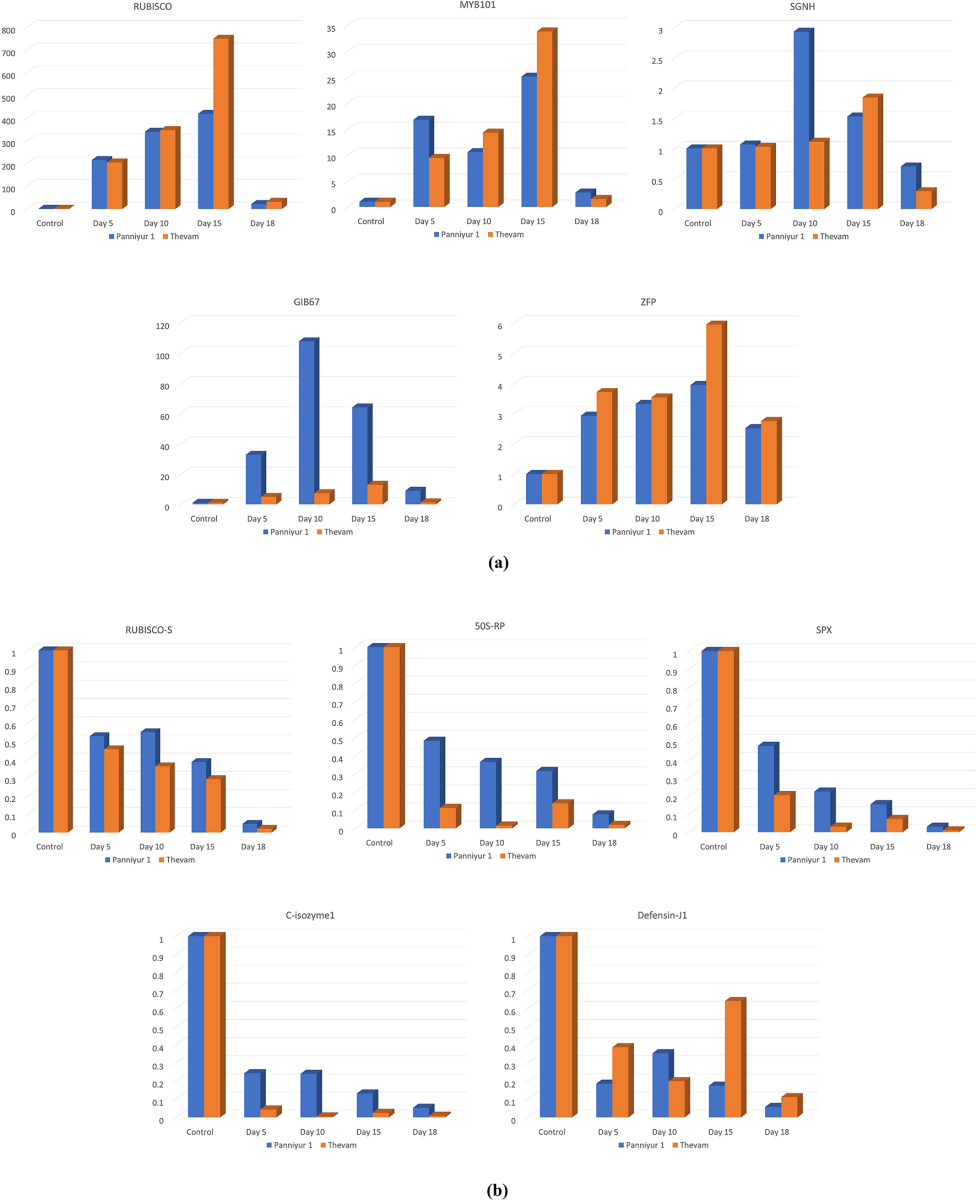
qPCR validation result of top five DEGs **(a)** upregulated **(b)** downregulated.

All five upregulated genes from the transcriptome exhibited higher expression in drought tolerant IISR Thevam compared to susceptible Panniyur 1 (**Figure 8**). The peak expression levels occurred on the 15th day of drought treatment, after which all gene expressions began to decrease by the 18th day. For *RUBISCO_S*, the highest expression was observed in the 15th day sample of IISR Thevam, with a 749.6-fold increase, followed by the 15th day sample of Panniyur 1, which showed a 418.76-fold increase. Similarly, *50S-RP* displayed its highest expression in the 15th day samples, with a 33.82-fold increase in IISR Thevam and a 25.1-fold increase in Panniyur 1.

For *SPX*, the expression was higher compared to the control, with the highest expression observed on the 10th day in Panniyur 1 (2.92-fold), followed by the 15th day in IISR Thevam (1.84-fold). In the case of *C-isozyme1*, the highest expression was recorded on the 10th day in Panniyur 1 (107.63-fold), followed by the 15th day in the same genotype (64-fold). For *Defensin-J1*, the highest expression was in IISR Thevam on the 15th day (5.94-fold), while Panniyur 1 showed a 3.94-fold increase. Expression of *RUBISCO-S* was found to be consistent in the transcriptome as well as qPCR analysis and is an ideal target to incorporate in the study of stress mechanism. The *ZAT10* gene, encoding a C2H2-EAR zinc finger protein, plays a critical role in plant responses to abiotic stresses, including drought.

The downregulated genes—*RUBISCO*, *MYB101*, *SGNH*, *GIB67* and *ZFP*—had maximum expression levels of 0.55, 0.48, 0.47, 0.24 and 0.64-fold, respectively (**Figure 8**). The study indicates the uniform expression patterns of genes in drought tolerance in two tolerant genotypes of black pepper, IISR-Thevam and Accession 4226 by low and high throughput methods.

## 4 Discussion

Black pepper is highly susceptible to drought stress, which significantly impacts its growth, yield, and secondary metabolite production. This study provides crucial insights into the molecular and physiological adaptations of black pepper under drought stress, highlighting the importance of transcriptomic changes and genetic variations in drought tolerance. Leaf biochemical parameters revealed an adaptive response to drought. To cope with water scarcity, the plant employs various physiological and molecular mechanisms, including the activation of drought-responsive genes, transcription factors, and antioxidant defence pathways. The decline in relative water content after 18 days of water deficit suggests a moderate level of dehydration tolerance in black pepper.

**Figure 9:**
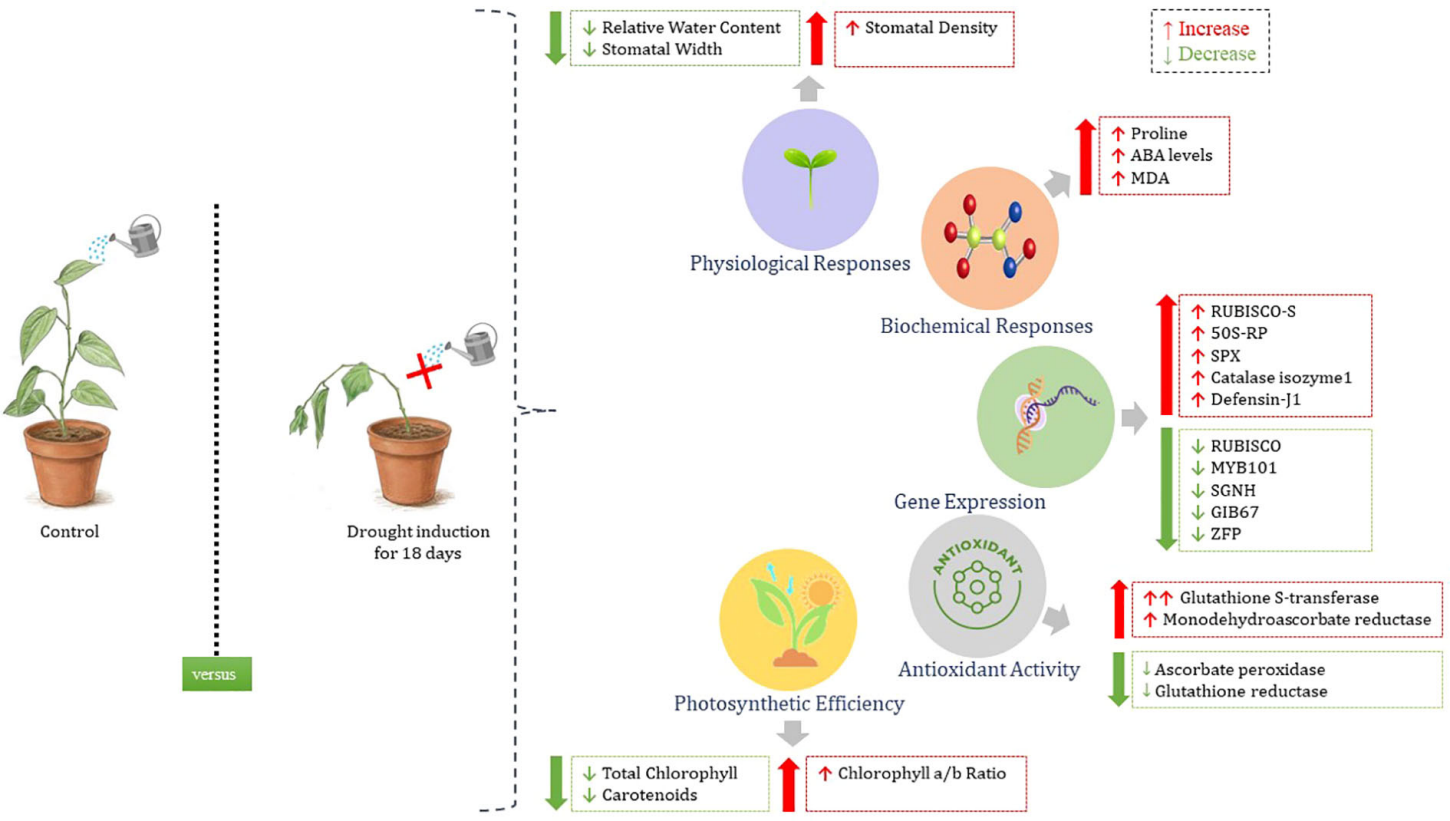
Schematic comparison of the responses of Accession 4226 to drought at physiological, biochemical and molecular level.

Under drought stress, significant physiological and biochemical changes were observed in the drought-tolerant black pepper accession 4226. Relative water content (RWC) declined from 91.2% to 80.0%, relatively low decline, indicating a better dehydration tolerance and an effective water conservation mechanisms (Farooq et al., 2009). Stomatal density remained relatively high (61.2 stomata/10x field), but reduced stomatal width during water stress (**Figure 2**) may aid in controlled transpiration under stress. The presence of leaf wax (3.7 µg/cm²) may also provide cuticular protection for water conservation (Kosma et al., 2009). Proline content more than doubled (65.3 to 151.5 µg/g), highlighting its role in osmotic adjustment and cellular protection under stress conditions (Ashraf and Foolad, 2007). Increase in soluble sugar content from 1.5 to 1.7% during water stress also contributes significantly to osmotic adjustment (REF). It has also been observed (though not studied here) that total amino acids significantly increased (Krishnamurthy et al., 2016) under water stress in black pepper which again points to osmatic adjustment. A decrease in total chlorophyll from 2.7 to 2.1 mg/g, alongside an increase in the Chl a/b ratio (3.3 to 4.2), suggests selective degradation of chlorophyll b and preservation of photosynthetic efficiency (Anjum et al., 2011). Meanwhile, a noticeable rise in MDA levels (21.3 to 53.8 µg/g) reflected increased oxidative stress, but elevated GST activity (from 41.0 to 822.0 units) suggests an efficient detoxification response (Gill and Tuteja, 2010). ABA levels also showed a mild increase (25.5 to 29.8 ppb), indicating enhanced drought signalling (Cutler et al., 2010). While total sugars and MDHAR levels increased modestly, glutathione reductase activity declined markedly (232.6 to 72.6 units), pointing towards a shift in redox balance and possible prioritization of alternate antioxidant pathways (Noctor et al., 2012). These cumulative adjustments emphasize the complex but coordinated response of black pepper to drought, involving water conservation, osmoprotection, hormone signalling, and ROS detoxification mechanisms, though abscisic acid mediated stress tolerance seems to have a limited role as indicated by very mild increase in ABA levels during stress and the same has been noticed in our earlier studies also. The schematic representation of the combined activity of the various responses is depicted in **Figure 9**.

The presence of differentially expressed genes (DEGs) in drought-treated plants indicates the activation of protective mechanisms, such as stress-responsive transcription factors, osmoprotectant synthesis, antioxidant pathways and signal transduction processes. The role of each gene and their significance in the mechanism of drought response is described below. These mechanisms collectively help to mitigate the adverse effects of water scarcity through complex regulatory networks. KEGG pathway analysis of DEGs has identified key metabolic and signalling pathways activated during drought stress, shedding light on molecular interactions and functional adaptations. Similarly, Gene Ontology (GO) classification categorizes drought-responsive genes based on biological processes, molecular functions, and cellular components, further aiding in the understanding of their specific roles in stress tolerance. Among the 442 stress-related and characterized genes identified from the transcriptome, distinct categories of genes contributing to drought stress were classified as given below.

### 4.1 Drought perception and initial response

The initial perception of drought stress in plants involves rapid signalling events mediated by abscisic acid (ABA) accumulation, reactive oxygen species (ROS) production, and phosphatase activity. These processes collectively trigger stomatal closure, activate stress-responsive genes, and initiate downstream protective mechanisms to mitigate water loss and cellular damage. Osmotic adjustment also plays a key role in stress tolerance. The high stomatal density as observed in Accession 4226 may not be a desirable trait under water stress but drastic reduction in stomatal width under water stress helps to conserve water, potentially aiding in water-use efficiency and mitigating excessive water loss under drought conditions. The rise in abscisic acid (ABA) levels from 23 to 29.8 ppb though suggests enhanced drought signalling, it indicates that ABA may not have a major role in stress response, rather osmotic adjustment and anti oxidant defense mechanism control drought tolerance. ABA serves as a central signalling molecule in drought-induced stomatal regulation, with genes such as CYP707A2 playing a role in ABA catabolism (Mega et al., 2015). EDR1, encoding a CTR1-like kinase, negatively regulates stress responses, while CIPK1 suppresses ABA signalling, thereby limiting drought tolerance (You et al., 2023)). Other regulatory elements, including ADC, NAC5, and WRKY60, contribute to osmotic adjustment, stress-related gene expression, and ABA signalling modulation (Shinozaki et al., 2003; Chen et al., 2010; Song et al., 2011). The role of protein phosphatases, including PP2C family members, further underscores the intricate regulation of drought responses (Hussain et al., 2017).

### 4.2 Osmoregulation

The significant increase in proline content from 65.3 to 151.5 µg/g highlights its role as a key osmoprotectant, aiding in cellular osmotic adjustment under drought stress. The increase in soluble sugar content also contribute to osmotic adjustment. The role of sugar alcohols such as mannitol and sorbitol in stress tolerance is well documented. AGPase plays a key role in starch biosynthesis, with differential regulation across tissues under drought conditions, while BAM1 contributes to transitory starch breakdown to support proline biosynthesis (Zanella et al., 2016). The NF-Y transcription factor enhances root growth and drought resistance by modulating indoleacetic acid transport via an ABA-mediated pathway (Han et al., 2020; Zhou et al., 2020). In accession 4226, upregulation of PDH, involved in proline catabolism, aligns with stress response patterns observed in other drought-tolerant plants (Furlan et al., 2020). Additionally, the accumulation of ATHB12 mRNA under drought stress and ABA treatment mirrors upregulation patterns in the transcriptome (Lee and Chun, 1998). Glycogen synthase kinases (GSKs/SKs), which exhibit distinct expression patterns under abiotic stress conditions, were under-expressed in the black pepper transcriptome, suggesting their role in stress adaptation (Zolkiewicz and Gruszka, 2022).

### 4.3 Antioxidant defense and metabolic adjustments

Drought stress triggers metabolic and antioxidant responses that counteract oxidative damage and maintain cellular stability. The substantial increase in malondialdehyde (MDA) levels from 21.3 to 53.8 µg/g indicates heightened oxidative stress under drought conditions. However, the activation of antioxidant defence mechanisms as indicated by increased activities of GST and MDHAR and the maintenance of ascorbate peroxidase activity likely helped in mitigating oxidative damage, maintaining cellular integrity despite the stress. The flavonoid biosynthesis pathway plays a crucial role in antioxidant defence, with genes such as anthocyanidin 3-O-glucosyltransferase, UDP-glycosyltransferase 73C5, and RHM1 showing downregulation, suggesting a shift in metabolic priorities (Oka et al., 2007; Tanaka et al., 2008; Yonekura-Sakakibara et al., 2019). Lignin biosynthesis, a key factor in drought adaptation, is modulated through the activity of caffeic acid 3-O-methyltransferase and phenylalanine ammonia-lyase, with changes in expression indicating potential shifts toward structural fortification, specifically, thicker or more lignified cell walls as part of the plant’s adaptive response to water deficit (Boerjan et al., 2003; Vogt, 2009). Terpenoid biosynthesis, particularly through germacrene D synthase, is upregulated, highlighting its role in antimicrobial defence and drought adaptation (Wang and Huo, 2022). The significant increase in glutathione S-transferase (GST) activity underscores its crucial role in detoxification and oxidative stress management under drought conditions, aiding in cellular protection and stress tolerance. The decline in glutathione reductase (GR) activity suggests a potential shift in redox homeostasis, which may impact the plant’s ability to regenerate reduced glutathione and maintain oxidative stress balance under drought conditions (Foyer and Noctor, 2005).

### 4.4 Energy production and metabolic regulation

Drought stress significantly alters energy production pathways, influencing key metabolic processes. Pyruvate metabolism, a critical link between glycolysis and the TCA cycle, is affected by the upregulation of genes such as probable sodium/metabolite cotransporter BASS2 and enolase, suggesting enhanced energy mobilization (Plaxton, 2004). In contrast, the downregulation of phosphoenolpyruvate carboxylase 2 and glycerate dehydrogenase indicates shifts in carbon fixation and photorespiratory adjustments (Chen et al., 2017). Fatty acid metabolism, a key alternative energy source, is modulated through lysophospholipid acyltransferase and enoyl-CoA hydratase/isomerase, highlighting increased lipid metabolism to compensate for energy deficits (Theodoulou and Eastmond, 2012). Additionally, the regulation of nucleotide metabolism, particularly through genes such as mediator of RNA polymerase II transcription subunit 15a and adenylate kinase 4, plays a role in maintaining ATP homeostasis and transcriptional regulation under stress (Bennett et al., 2020).

### 4.5 Structural and physiological adaptations

Drought stress leads to structural and physiological adaptations that help plants maintain integrity under water-limiting conditions. Drought stress led to a decline in total chlorophyll content, suggesting a reduction in photosynthetic capacity. However, the increase in the chlorophyll a/b ratio indicates the selective degradation of chlorophyll b, which may help optimize light absorption under stress conditions. Chlorophyll metabolism is significantly affected, with protochlorophyllide reductase and chlorophyllide a oxygenase upregulated, suggesting an adaptive response to regulate chlorophyll biosynthesis and maintain photosynthetic efficiency (Kobayashi et al., 2013). Photosystem I subunit O and NDH subunit of subcomplex B 2 are upregulated, enhancing cyclic electron flow and energy generation during drought stress (Yamori et al., 2011). The fall in carotenoid levels suggest a potential reduction in photoprotection but the decrease is not significant, suggesting better photoprotection as under normal conditions. Cuticle and wax biosynthesis are modulated, with genes such as *CER3* and 3-ketoacyl-CoA synthase downregulated, indicating a potential reduction in cuticle reinforcement and increased water loss (Kosma et al., 2009) but the cuticular wax may still regulate water loss during drought stress. Additionally, cell wall remodelling is evident, with upregulation of genes such as endo-1,4-beta-xylanase 1 and pectin methylesterase 61, suggesting modifications in cell wall composition to enhance stress resilience (Wolf et al., 2012). Lysosome-mediated protein degradation, indicated by upregulated protease inhibitor precursor expression, plays a crucial role in maintaining protein homeostasis (Moloi and Ngara, 2023). The reduction in stomatal width during drought is an advantageous trait, indicating a more efficient stomatal closure mechanism. This helps minimize water loss through transpiration, thereby conserving water for essential metabolic processes, thus maintaining growth under water stress.

### 4.6 Protein stability and stress recovery

In addition to gene expression changes and signaling pathways that mediate drought responses, the stability and proper functioning of proteins under stress conditions are equally critical. Drought stress can cause protein denaturation, misfolding, and degradation, which compromise cellular homeostasis and recovery (Kosová et al., 2011). Therefore, maintaining protein stability and enabling efficient post-stress recovery mechanisms represent another crucial layer of drought tolerance. Chaperones and heat shock proteins (HSPs) play a crucial role in maintaining protein stability under drought stress by assisting in protein folding, preventing aggregation, and facilitating stress recovery. The upregulation of chaperone protein DnaJ 20 and AAA-ATPase At5g57480 suggests an increased demand for molecular chaperones to mitigate protein misfolding and degradation (Chen et al., 2004). Similarly, chaperone protein DnaJ GFA2 and CHAPERONE-LIKE PROTEIN OF POR1 exhibit increased expression, indicating a role in proteostasis maintenance in chloroplasts and mitochondria (Christensen et al., 2002). The downregulation of DnaJ 8 and other members of the DnaJ family suggests functional specialization in drought response mechanisms (Zhou et al., 2012). Heat shock proteins such as Hsp20/alpha crystallin family protein are upregulated, reinforcing their role in stabilizing proteins against drought-induced denaturation (Sun et al., 2002). Collectively, these findings highlight the dynamic role of chaperones, heat shock proteins, and protein-protein interactions in enhancing black pepper’s resilience to drought stress.

## 5 Conclusion

This study highlights the complex molecular mechanisms and physiological adaptations in black pepper (*Piper nigrum* L.) under drought stress, focusing on the comparative analysis of contrasting genotypes, IISR Thevam and Accession 4226 against Panniyur 1. By employing transcriptomic and gene expression analyses, we identified key differentially expressed genes (DEGs) that play critical roles in stress tolerance. Notably, IISR Thevam exhibited higher expression of drought-responsive genes, including *RUBISCO-S*, *50S-RP*, *SPX* and *Defensin-J1*, which are associated with photosynthetic carbon assimilation, drought-induced regulation of ribosomal protein synthesis, phosphate signaling and homeostasis under water deficit conditions, and stress-responsive antimicrobial defense, respectively. The contrasting performance of IISR Thevam and Panniyur 1 underlines the genetic basis of drought tolerance in black pepper and reinforces the significance of genetic diversity in breeding programs. The superior drought adaptability of IISR Thevam, as evidenced by sustained gene expression and enhanced physiological responses, positions it as a valuable resource for developing drought-resilient varieties. Overall, this study provides foundational insights into the molecular basis of drought stress tolerance in black pepper. The identified genes and pathways serve as potential targets for genetic improvement, facilitating the development of high-yielding, drought-tolerant cultivars to ensure sustainable black pepper production amidst the challenges posed by climate change and are available at http://14.139.189.24/transcriptome/.

## Data Availability

The datasets presented in this study can be found in online repositories. The names of the repository/repositories and accession number(s) can be found in the article/**Supplementary Material**.

## References

[B1] Ambrozim C. S. Medici L. O. da Cruz E. S. Abreu J. F. G. de Carvalho D. F. (2022). Physiological response of black pepper (Piper nigrum L.) to deficit irrigation. Rev. Ciec. Agron. 53, e20207348. doi: 10.5935/1806-6690.20220002

[B2] Anjum S. A. Xie X.-Y. Wang L.-C. Saleem M. F. Man C. Lei W. (2011). Morphological, physiological and biochemical responses of plants to drought stress. Afr J. Agric. Res. 6, 2026–2032. doi: 10.5897/AJAR10.027

[B3] Ashraf M. Foolad M. R. (2007). Roles of glycine betaine and proline in improving plant abiotic stress resistance. Environ. Exp. Bot. 59, 206–216. doi: 10.1016/J.ENVEXPBOT.2005.12.006

[B4] Babu Paul B. Mathew D. Beena S. Shylaja M. R. (2019). Comparative transcriptome analysis reveals the signal proteins and defence genes conferring foot rot (Phytophthora capsici sp. nov.) resistance in black pepper (Piper nigrum L.). Physiol. Mol. Plant Pathol. 108, 101436. doi: 10.1016/J.PMPP.2019.101436

[B5] Bates L. S. Waldren R. P. Teare I. D. (1973). Rapid determination of free proline for water-stress studies. Plant Soil 39, 205–207. doi: 10.1007/BF00018060

[B6] Bennett N. K. Nguyen M. K. Darch M. A. Nakaoka H. J. Cousineau D. ten Hoeve J. . (2020). Defining the ATPome reveals cross-optimization of metabolic pathways. Nat. Commun. 11, 4319. doi: 10.1038/S41467-020-18084-6, PMID: 32859923 PMC7455733

[B7] Boerjan W. Ralph J. Baucher M. (2003). Lignin Biosynthesis. Annu. Rev. Plant Biol. 54, 519–546. doi: 10.1146/ANNUREV.ARPLANT.54.031902.134938/1 14503002

[B8] Butt M. S. Pasha I. Sultan M. T. Randhawa M. A. Saeed F. Ahmed W. (2013). Black pepper and health claims: a comprehensive treatise. Crit. Rev. Food Sci. Nutr. 53, 875–886. doi: 10.1080/10408398.2011.571799, PMID: 23768180

[B9] Carlberg I. Mannervik B. (1985). Glutathione reductase. Methods Enzymol. 113, 484–490. doi: 10.1016/S0076-6879(85)13062-4, PMID: 3003504

[B10] Chen G. Cui J. Wang L. Zhu Y. Lu Z. Jin B. (2017). Genome-wide identification of circular RNAs in Arabidopsis thaliana. Front. Plant Sci. 8. doi: 10.3389/FPLS.2017.01678/BIBTEX PMC5623955 29021802

[B11] Chen H. Lai Z. Shi J. Xiao Y. Chen Z. Xu X. (2010). Roles of arabidopsis WRKY18, WRKY40 and WRKY60 transcription factors in plant responses to abscisic acid and abiotic stress. BMC Plant Biol. 10, 1–15. doi: 10.1186/1471-2229-10-281/FIGURES/10, PMID: 21167067 PMC3023790

[B12] Chen Q. Weng Q. YuanChao W. Zheng X. (2004). Identification and sequencing of ribosomal DNA-ITS of Phytophthora sojae in Fujian. Acta Phytopathologica Sin. 34, 112–116.

[B13] Christensen C. A. Gorsich S. W. Brown R. H. Jones L. G. Brown J. Shaw J. M. . (2002). Mitochondrial GFA2 is required for synergid cell death in Arabidopsis. Plant Cell 14, 2215–2232. doi: 10.1105/TPC.002170, PMID: 12215516 PMC150766

[B14] Cutler S. R. Rodriguez P. L. Finkelstein R. R. Abrams S. R. (2010). Abscisic acid: emergence of a core signaling network. Annu. Rev. Plant Biol. 61, 651–679. doi: 10.1146/ANNUREV-ARPLANT-042809-112122, PMID: 20192755

[B15] Das P. Sheeja T. E. Saha B. Fayad A. Chandra T. Angadi U. B. . (2025). Genome-wide identification of copy number variation in diverse black pepper accessions. Planta 261, 81. doi: 10.1007/S00425-025-04658-5, PMID: 40057659

[B16] De Pascale S. Ruggiero C. Barbieri G. Maggio A. (2003). Physiological responses of pepper to salinity and drought. J. Am. Soc. Hortic. Sci. 128, 48–54. doi: 10.21273/JASHS.128.1.0048

[B17] Farooq M. Wahid A. Kobayashi N. Fujita D. Basra S. M. A. (2009). Plant drought stress: effects, mechanisms and management. Agron. Sustain. Dev. 29, 1 29, 185–212. doi: 10.1051/AGRO:2008021

[B18] Ferreira T. R. Sallin V. P. Cerri Neto B. Crasque J. Pires A. Rodrigues P. S. . (2024). Morphophysiological responses of black pepper to recurrent water deficit. Photosynthetica. 62, 292–301. doi: 10.32615/PS.2024.030, PMID: 39649363 PMC11622609

[B19] Foyer C. H. Noctor G. (2005). Redox homeostasis and antioxidant signaling: A metabolic interface between stress perception and physiological responses. Plant Cell 17, 1866–1875. doi: 10.1105/TPC.105.033589, PMID: 15987996 PMC1167537

[B20] Furlan A. L. Bianucci E. Giordano W. Castro S. Becker D. F. (2020). Proline metabolic dynamics and implications in drought tolerance of peanut plants. Plant Physiol. Biochem. 151, 566–578. doi: 10.1016/J.PLAPHY.2020.04.010, PMID: 32320942

[B21] George K. J. Malik N. Vijesh Kumar I. P. Krishnamurthy K. S. (2017). Gene expression analysis in drought tolerant and susceptible black pepper (Piper nigrum L.) in response to water deficit stress. Acta Physiol. Plant 39, 1–9. doi: 10.1007/S11738-017-2398-5/FIGURES/3

[B22] Gill S. S. Tuteja N. (2010). Reactive oxygen species and antioxidant machinery in abiotic stress tolerance in crop plants. Plant Physiol. Biochem. 48, 909–930. doi: 10.1016/J.PLAPHY.2010.08.016, PMID: 20870416

[B23] González L. González-Vilar M. (2001). “ Determination of relative water content,” in Handbook of plant ecophysiology techniques ed. Reigosa Roger M. J. (Dordrecht: Springer), 207–212. doi: 10.1007/0-306-48057-3_14

[B24] Gonzalez-Dugo V. Durand J.-L. Gastal F. (2010). Water deficit and nitrogen nutrition of crops. A review Water deficit and nitrogen nu-trition of crops. A review. Agronomy for Sustainable Development Water deficit and nitrogen nutrition of crops. A review. Agron. Sustain. Dev. 30, 529–544. doi: 10.1051/agro/2009059

[B25] Griffith O. W. (1980). Determination of glutathione and glutathione disulfide using glutathione reductase and 2-vinylpyridine. Anal. Biochem. 106, 207–212. doi: 10.1016/0003-2697(80)90139-6, PMID: 7416462

[B26] Han M. L. Yin J. Zhao Y. H. Sun X. W. Meng J. X. Zhou J. . (2020). How the color fades from malus halliana flowers: transcriptome sequencing and DNA methylation analysis. Front. Plant Sci. 11. doi: 10.3389/FPLS.2020.576054, PMID: 33072152 PMC7539061

[B27] Hossain M. A. Araki H. Takahashi T. (2011). Poor grain filling induced by waterlogging is similar to that in abnormal early ripening in wheat in Western Japan. Field Crops Res. 123, 100–108. doi: 10.1016/J.FCR.2011.05.005

[B28] Hu L. Hao C. Fan R. Wu B. Tan L. Wu H. (2015). *De novo* assembly and characterization of fruit transcriptome in black pepper (Piper nigrum). PloS One. 10, e0136028. doi: 10.1371/journal.pone.0129822, PMID: 26121657 PMC4488137

[B29] Hu L. Xu Z. Wang M. Fan R. Yuan D. Wu B. . (2019). The chromosome-scale reference genome of black pepper provides insight into piperine biosynthesis. Nat. Commun. 10, 4702. doi: 10.1038/s41467-019-12607-6, PMID: 31619678 PMC6795880

[B30] Hussain Z. Chaudhri V. K. Pandey A. Khan R. Srivastava A. K. Maurya R. (2017). Isolation and evaluation of piperine from black pepper and white pepper. Available online at: www.wjpps.com.

[B31] Karkacier M. Erbas M. Uslu M. K. Aksu M. (2003). Comparison of different extraction and detection methods for sugars using amino-bonded phase HPLC. J. Chromatogr Sci. 41, 331–333. doi: 10.1093/chromsci/41.6.331, PMID: 12935307

[B32] Kobayashi K. Sasaki D. Noguchi K. Fujinuma D. Komatsu H. Kobayashi M. . (2013). Photosynthesis of root chloroplasts developed in arabidopsis lines overexpressing GOLDEN2-LIKE transcription factors. Plant Cell Physiol. 54, 1365–1377. doi: 10.1093/pcp/pct086. K Kobayashi, D Sasaki, K Noguchi, D Fujinuma, H Komatsu, M Kobayashi, M Sato., PMID: 23749810 PMC3730084

[B33] Kosma D. K. Bourdenx B. Bernard A. Parsons E. P. Lü S. Joubès J. . (2009). The impact of water deficiency on leaf cuticle lipids of arabidopsis. Plant Physiol. 151, 1918–1929. doi: 10.1104/PP.109.141911, PMID: 19819982 PMC2785987

[B34] Kosová K. Vítámvás P. Prášil I. T. Renaut J. (2011). Plant proteome changes under abiotic stress — Contribution of proteomics studies to understanding plant stress response. J. Proteomics 74, 1301–1322. doi: 10.1016/J.JPROT.2011.02.006, PMID: 21329772

[B35] Krishnamurthy K. S. Ankegowda S. J. (1998). Impact of water stress on some physiological parameters in black pepper. Calicut.

[B36] Krishnamurthy K. S. ANKEGOWDA S. J. SAJI K. V. (2000). Water Stress Effects on Membrane Damage and Activities of Catalase, Peroxidase and Superoxide Dismutase Enzymes in Black Pepper (Piper nigrum L.). Journal of Plant Biology 27, 39–42.

[B37] Krishnamurthy K. George J. K. Ankagoe S. Umadev P. (2016). Black pepper and water stress 321–332. doi: 10.1007/978-81-322-2725-0

[B38] Krishnamurthy K. Kandiannan K. Sibin C. Chempakam B. Ankegowda S. (2011). Trends in climate and productivity and relationship between climatic variables and productivity in black pepper (Piper nigrum). Indian J. Agric. Sci. 81. Available at: https://epubs.icar.org.in/index.php/IJAgS/article/view/8436 (Accessed April 8, 2025).

[B39] Krishnamurthy K. S. Saji K. V. (2006). Response of Piper species to water stress. Indian J. Horticulture 63, 433–438.

[B40] Lau E. T. Khew C. Y. Hwang S. S. (2020). Transcriptomic analysis of pepper plants provides insights into host responses to Fusarium solani infestation. J. Biotechnol. 314–315, 53–62. doi: 10.1016/J.JBIOTEC.2020.03.014, PMID: 32302654

[B41] Lee Y. H. Chun J. Y. (1998). A new homeodomain-leucine zipper gene from Arabidopsis thaliana induced by water stress and abscisic acid treatment. Plant Mol. Biol. 37, 377–384. doi: 10.1023/A:1006084305012, PMID: 9617808

[B42] Lichtenthaler H. K. (1987). 34] Chlorophylls and carotenoids: Pigments of photosynthetic biomembranes. Methods Enzymol. 148, 350–382. doi: 10.1016/0076-6879(87)48036-1

[B43] Livak K. J. Schmittgen T. D. (2001). Analysis of relative gene expression data using real-time quantitative PCR and the 2-ΔΔCT method. Methods 25, 402–408. doi: 10.1006/meth.2001.1262, PMID: 11846609

[B44] Mega R. Meguro-Maoka A. Endo A. Shimosaka E. Murayama S. Nambara E. . (2015). Sustained low abscisic acid levels increase seedling vigor under cold stress in rice (Oryza sativa L.). Sci. Rep. 5, 1 5, 1–1 5,13. doi: 10.1038/srep13819, PMID: 26350634 PMC4563555

[B45] Mir R. R. Zaman-Allah M. Sreenivasulu N. Trethowan R. Varshney R. K. (2012). Integrated genomics, physiology and breeding approaches for improving drought tolerance in crops. Theor. Appl. Genet. 125, 625–645. doi: 10.1007/S00122-012-1904-9, PMID: 22696006 PMC3405239

[B46] Moloi S. J. Ngara R. (2023). The roles of plant proteases and protease inhibitors in drought response: a review. Front. Plant Sci. 14. doi: 10.3389/FPLS.2023.1165845, PMID: 37143877 PMC10151539

[B47] Nakano Y. Asada K. (1981). Hydrogen peroxide is scavenged by ascorbate-specific peroxidase in spinach chloroplasts. Plant Cell Physiol. 22, 867–880. doi: 10.1093/oxfordjournals.pcp.a076232

[B48] Negi A. George Kokkat J. Jasrotia R. S. Madhavan S. Jaiswal S. Angadi U. B. . (2021). Drought responsiveness in black pepper (Piper nigrum L.): Genes associated and development of a web-genomic resource. Physiol. Plant 172, 669–683. doi: 10.1111/PPL.13308, PMID: 33305409

[B49] Noctor G. Mhamdi A. Chaouch S. Han Y. Neukermans J. Marquez-Garcia B. . (2012). Glutathione in plants: an integrated overview. Plant Cell Environ. 35, 454–484. doi: 10.1111/J.1365-3040.2011.02400.X, PMID: 21777251

[B50] Oka T. Nemoto T. Jigami Y. (2007). Functional analysis of Arabidopsis thaliana RHM2/MUM4, a multidomain protein involved in UDP-D-glucose to UDP-L-rhamnose conversion. J. Biol. Chem. 282, 5389–5403. doi: 10.1074/JBC.M610196200, PMID: 17190829

[B51] Plaxton W. C. (2004). “ Principles of metabolic control,” in Functional metabolism. Ed. Storey K. B. ( John Wiley & Sons, Ltd), 1–24. doi: 10.1002/047167558X.CH1

[B52] Prakash K. M. Joseph J. Santhoshkumar A. V. Saji K. V. Puthiamadom N. Ramachandran A. (2023). Drought response of biotic-stress tolerant accessions of black pepper (Piper nigrum L.). J. Trop. Agric. 61, 189–195.

[B53] Quarrie S. A. Whitford P. N. Appleford N. E. J. Wang T. L. Cook S. K. Henson I. E. . (1988). A monoclonal antibody to (S)-abscisic acid: its characterisation and use in a radioimmunoassay for measuring abscisic acid in crude extracts of cereal and lupin leaves. Planta 173, 330–339. doi: 10.1007/BF00401020/METRICS, PMID: 24226540

[B54] Ramadasan A. Vasantha S. (1994). Environmental stress reaction of black pepper. Spice India 7, 12–15.

[B55] Reich P. B. (1984). Leaf stomatal density and diffusive conductance in three amphistomatous hybrid poplar cultivars. New Phytol. 98, 231–239. doi: 10.1111/J.1469-8137.1984.TB02733.X

[B56] Schreiber L. Schönherr J. (1993). Mobilities of organic compounds in reconstituted cuticular wax of barley leaves: Determination of diffusion coefficients. Pestic Sci. 38, 353–361. doi: 10.1002/PS.2780380413

[B57] Shabala S. N. Shabala S. I. Martynenko A. I. Babourina O. Newman I. A. (1998). Salinity effect on bioelectric activity, growth, Na+ accumulation and chlorophyll fluorescence of maize leaves: a comparative survey and prospects for screening. Funct. Plant Biol. 25, 609–616. doi: 10.1071/PP97146

[B58] Shinozaki K. Yamaguchi-Shinozaki K. Seki M. (2003). Regulatory network of gene expression in the drought and cold stress responses. Curr. Opin. Plant Biol. 6, 410–417. doi: 10.1016/S1369-5266(03)00092-X, PMID: 12972040

[B59] Sivaraman K. Kandiannan K. Peter K. V. Thankamani C. K. (1999). Agronomy of black pepper (Piper nigrum L.) - a review. J. Spices Aromatic Crops 8, 01–18.

[B60] Song S. Y. Chen Y. Chen J. Dai X. Y. Zhang W. H. (2011). Physiological mechanisms underlying OsNAC5-dependent tolerance of rice plants to abiotic stress. Planta 234, 331–345. doi: 10.1007/S00425-011-1403-2, PMID: 21448719

[B61] Sozzi G. O. Peter K. V. Nirmal Babu K. Divakaran M. (2012). “ Capers and caperberries,” in Handbook of herbs and spices: second edition, 2, edd. Peter K. V. Peter K. V. (England: Woodhead Publishing), 193–224. doi: 10.1533/9780857095688.193

[B62] Sreekumar S. Divya K. Joy N. Soniya E. V. (2022). *De novo* transcriptome profiling unveils the regulation of phenylpropanoid biosynthesis in unripe Piper nigrum berries. BMC Plant Biol. 22, 501. doi: 10.1186/S12870-022-03878-1, PMID: 36284267 PMC9597958

[B63] Sun W. Van Montagu M. Verbruggen N. (2002). Small heat shock proteins and stress tolerance in plants. Biochimica et Biophysica Acta (BBA). - Gene Structure Expression 1577, 1–9. doi: 10.1016/S0167-4781(02)00417-7, PMID: 12151089

[B64] Tanaka Y. Sasaki N. Ohmiya A. (2008). Biosynthesis of plant pigments: anthocyanins, betalains and carotenoids. Plant J. 54, 733–749. doi: 10.1111/J.1365-313X.2008.03447.X, PMID: 18476875

[B65] Thankamani C. K. Thankamani C. K. Ashokan P. K. (2002). ). Chlorophyll and leaf epicuticular wax contents of black pepper (Piper nigrum) varieties in response to water stress. J. Medicinal Aromatic Plant Sci. 24, 943–946. doi: 10.0/FONT/BOOTSTRAP-ICONS.MIN.CSS

[B66] Thankamani C. K. Thankamani C. K. Chempakam B. Ashokan P. K. (2003). Water stress induced changes in enzyme activities and lipid peroxidation in black pepper (Piper nigrum). J. Medicinal Aromatic Plant Sci. 25, 646–650. doi: 10.0/FONT/BOOTSTRAP-ICONS.MIN.CSS

[B67] Theodoulou F. L. Eastmond P. J. (2012). Seed storage oil catabolism: a story of give and take. Curr. Opin. Plant Biol. 15, 322–328. doi: 10.1016/J.PBI.2012.03.017, PMID: 22516438

[B68] Vasantha S. Thomas T. V. Ramadasan A. Zachariah T. J. (1990). Drought tolerance in Black pep per (Piper nigrum L.) cultivars: an evaluation of phys iological parameters - Google Search. Indian J. Plant Physiol. 33, 363–366.

[B69] Verma S. Dubey R. S. (2003). Lead toxicity induces lipid peroxidation and alters the activities of antioxidant enzymes in growing rice plants. Plant Sci. 164, 645–655. doi: 10.1016/S0168-9452(03)00022-0

[B70] Vijayakumari K. Purthur J. T. (2014). Drougt stress responses in tolerant and sensitive varieties of black pepper (Piper nigrum L.). J. Plantation Crops 42, 78–85.

[B71] Vogt T. (2009). Phenylpropanoid biosynthesis. Mol. Plant 3, 2–20. doi: 10.1093/mp/ssp106, PMID: 20035037

[B72] Wang N. Huo Y. X. (2022). Using genome and transcriptome analysis to elucidate biosynthetic pathways. Curr. Opin. Biotechnol. 75, 102708. doi: 10.1016/j.copbio.2022.102708, PMID: 35278747

[B73] Wolf S. Hématy K. Höfte H. (2012). Growth control and cell wall signaling in plants. Annu. Rev. Plant Biol. 63, 381–407. doi: 10.1146/ANNUREV-ARPLANT-042811-105449, PMID: 22224451

[B74] Yamori W. Sakata N. Suzuki Y. Shikanai T. Makino A. (2011). Cyclic electron flow around photosystem I via chloroplast NAD(P)H dehydrogenase (NDH) complex performs a significant physiological role during photosynthesis and plant growth at low temperature in rice. Plant J. 68, 966–976. doi: 10.1111/J.1365-313X.2011.04747.X, PMID: 21848656

[B75] Yonekura-Sakakibara K. Higashi Y. Nakabayashi R. (2019). The origin and evolution of plant flavonoid metabolism. Front. Plant Sci. 10. doi: 10.3389/FPLS.2019.00943/XML/NLM, PMID: 31428108 PMC6688129

[B76] You Z. Guo S. Li Q. Fang Y. Huang P. Ju C. . (2023). The CBL1/9-CIPK1 calcium sensor negatively regulates drought stress by phosphorylating the PYLs ABA receptor. Nat. Commun. 14, 1 14, 1–14. doi: 10.1038/s41467-023-41657-0, PMID: 37735173 PMC10514306

[B77] Zanella M. Borghi G. L. Pirone C. Thalmann M. Pazmino D. Costa A. . (2016). β-amylase 1 (BAM1) degrades transitory starch to sustain proline biosynthesis during drought stress. J. Exp. Bot. 67, 1819–1826. doi: 10.1093/JXB/ERV572, PMID: 26792489

[B78] Zhou Y. Zhang Y. Wang X. Han X. An Y. Lin S. . (2020). Root-specific NF-Y family transcription factor, PdNF-YB21, positively regulates root growth and drought resistance by abscisic acid-mediated indoylacetic acid transport in Populus. New Phytol. 227, 407–426. doi: 10.1111/NPH.16524, PMID: 32145071

[B79] Zhou W. Zhou T. Li M. X. Zhao C. L. Jia N. Wang X. X. . (2012). The Arabidopsis J-protein AtDjB1 facilitates thermotolerance by protecting cells against heat-induced oxidative damage. New Phytol. 194, 364–378. doi: 10.1111/J.1469-8137.2012.04070.X, PMID: 22356282

[B80] Zolkiewicz K. Gruszka D. (2022). Glycogen synthase kinases in model and crop plants – From negative regulators of brassinosteroid signaling to multifaceted hubs of various signaling pathways and modulators of plant reproduction and yield. Front. Plant Sci. 13. doi: 10.3389/FPLS.2022.939487/FULL, PMID: 35909730 PMC9335153

